# Factors influencing the discontinuation of biologic therapies in patients with ulcerative colitis

**DOI:** 10.1186/s40780-024-00386-2

**Published:** 2024-10-18

**Authors:** Arisa Fukuyama, Akio Nakashima, Motoyasu Miyazaki, Masakatsu Fujiki, Hideki Kakimoto, Takashi Hisabe, Osamu Imakyure

**Affiliations:** 1 Department of Pharmacy, Fukuoka University Chikushi Hospital, 1-1-1, Zokumyoin, Chikushino-Shi, Fukuoka, 818-8502 Japan; 2 Department of Pharmaceutical and Health Care Management, Faculty of Pharmaceutical Sciences, Fukuoka University, Nanakuma 8-19-1, Jonan-Ku, Fukuoka, 814-0180 Japan; 3 Department of Gastroenterology, Fukuoka University Chikushi Hospital, 1-1-1, Zokumyoin, Chikushino-Shi, Fukuoka, 818-8502 Japan

**Keywords:** Ulcerative colitis, Biological agent, Discontinuation

## Abstract

**Background:**

The therapeutic landscape for ulcerative colitis (UC) has recently broadened to include anti-TNFα, anti-integrin, and anti-IL-12/23p40 antibody agents. These biological agents are tailored to individual patient profiles. However, some patients cease biological treatment. This study investigates factors influencing the discontinuation of biological treatment in UC patients.

**Methods:**

This retrospective single-cohort study encompasses UC patients who commenced treatment with biological agents like infliximab, adalimumab, golimumab, vedolizumab, and ustekinumab from April 2019 to March 2022. Patients were categorized into continuation and discontinuation groups based on their one-year treatment status. Baseline characteristics were compared between the groups.

**Results:**

Of the 116 UC patients, 102 were included in the study. Among these, 74 (72.5%) continued and 28 (27.5%) discontinued biological therapy. Discontinuation rates for infliximab, adalimumab, golimumab, vedolizumab, and ustekinumab were 33.3%, 25.0%, 50.0%, 30.2%, and 15.6%, respectively. The primary discontinuation reason was lack of efficacy (85.7%), followed by adverse events (7.1%), pregnancy (3.6%), and death (3.6%). The discontinuation group had a significantly lower rate of concomitant thiopurine compared to the continuation group (28.6% *vs*. 56.8%, *p* = 0.0132). Multivariable analysis revealed that concomitant thiopurine was independently associated with therapy continuation (*p* = 0.0075).

**Conclusion:**

The study indicates that concomitant thiopurine significantly correlates with the continuation of biological therapies in UC patients, underscoring the importance of concomitant thiopurine in sustaining biological therapy. Further studies are warranted to assess the efficacy of combination therapy.

**Supplementary Information:**

The online version contains supplementary material available at 10.1186/s40780-024-00386-2.

## Background

Ulcerative colitis (UC) is a chronic inflammatory disease marked by symptoms such as rectal urgency, tenesmus, fecal incontinence, mucus discharge, nocturnal defecations, and abdominal pain. Its incidence is on the rise globally [1]. Traditional UC treatments have included 5-aminosalicylic acid (5-ASA) agents, corticosteroids, and thiopurines. However, recent years have seen the emergence of biological agents like anti-cytokines, anti-integrins, and Janus kinase inhibitors as promising alternatives [2–4].

Anti-TNFα drugs (e.g., infliximab [IFX], adalimumab [ADA], golimumab [GLM]), vedolizumab (VDZ), an anti-integrin antibody, and ustekinumab (UST), an anti-IL-12/23p40 antibody, have proven effective in inducing and maintaining remission in UC [5–10]. However, primary non-response has been observed in 10% to 40% of inflammatory bowel disease (IBD) patients treated with anti-TNF-α drugs [11]. Some patients also cease treatment with VDZ or UST due to inefficacy or adverse effects [12, 13]. Papamichael et al. reported that 55.6% of UC patients who discontinued IFX therapy due to primary non-response underwent colectomy [14], suggesting that discontinuation of biological therapy may impact future clinical outcomes.

While numerous studies have reported on the efficacy and safety of biological therapy, the factors influencing the discontinuation remain elusive. By considering patients’ backgrounds in treatment, the outcome of biological treatment could be enhanced. Therefore, identifying factors associated with biologic discontinuation can aid in optimizing biological therapy for UC. This study investigates the factors leading to discontinuation of biological therapies based on the clinical characteristics of the patients who used them.

## Methods

### Patients

This retrospective single-center cohort study was conducted at Fukuoka University Chikushi Hospital. It included UC patients who initiated IFX, ADA, GLM, VDZ, or UST between April 2019 and March 2022. Exclusions were patients with prior colectomy, those who initiated these biological agents at other hospitals, or those lost to follow-up or transferred within a year of initiation.

### Data collection

Demographic and clinical characteristics at the time of biologic initiation were collected from electronic medical records: sex, age, body mass index (BMI), disease duration, colonic area involved, concomitant medications (5-ASA, corticosteroids, thiopurines), therapy history (anti-TNFα agents, VDZ, UST, and Janus kinase (JAK) inhibitors), initiated biologics, C-reactive protein (CRP), and clinical disease activity score (partial Mayo score). The partial Mayo score, excluding the endoscopy subscore, comprised rectal bleeding, stool frequency, and physician assessment. Because the data were collected at the time of biologics initiation, patients who did not meet the criteria for moderate-to-severe disease severity were also included in this study.

### Definition

Patients were categorized into continuation and discontinuation groups based on whether they continued each biologic agent for one-year. Patients were classified as discontinuation if they received the biological agent beyond the washout period, defined as at least twice the usual dosing interval. Generally, IFX was administered on day 1 and at weeks 2, 6, and every 8 weeks thereafter; ADA every 2 weeks; GLM on day 1 and at week 2, and every 4 weeks thereafter; VDZ on day1 and at weeks 2, 6 and every 8 weeks thereafter; UST every 8 weeks.

### Statistical analysis

Baseline characteristics were compared between the continuation and discontinuation groups. Univariate logistic regression analysis was performed to identify factors associated with biologic discontinuation, calculating crude odds ratio (OR) and 95% confidence intervals (CI). Covariates with a *p*-value < 0.1 in the univariate analysis were included in the multivariate logistic regression model. In the comparison of clinical characteristics between discontinuation and continuation groups for each drug, categorical and continuous variables were analyzed using Fisher’s exact test and the Mann–Whitney U test, respectively. Statistical significance was defined as a *p*-value less than 0.05. All statistical analyses were conducted using JMP® Pro 17.2.0 (SAS Institute Inc., Cary, NC, USA).

## Results

### Patients

**Fig. 1:**
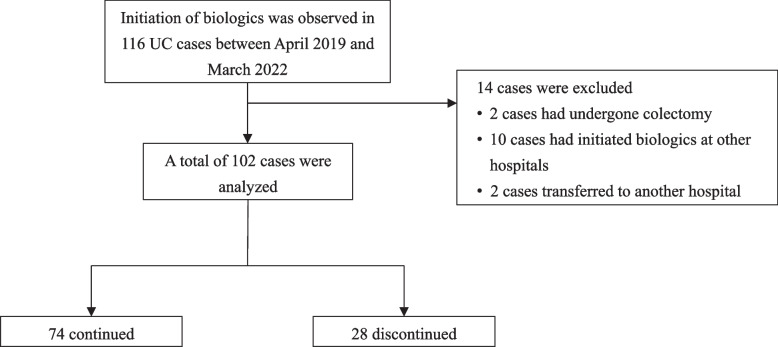
Flowchart of the study. *UC*, ulcerative colitis

**Fig. 2:**
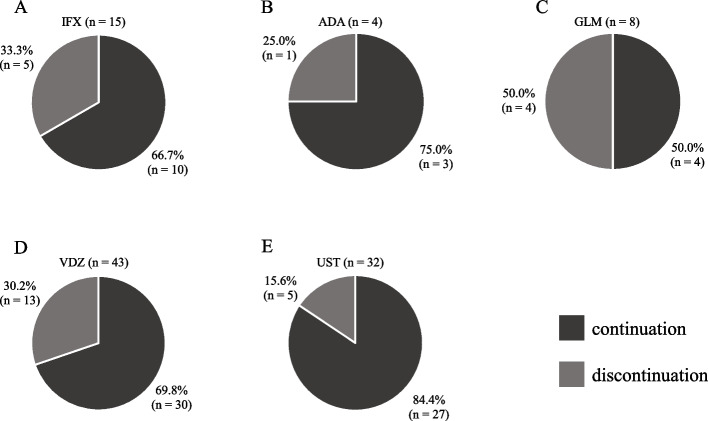
Discontinuation rates of IFX (**A**), ADA (**B**), GLM (**C**), VDZ (**D**), and UST (**E**). *IFX*, infliximab; *ADA*, adalimumab; *GLM*, golimumab; *VDZ*, vedolizumab; *UST*, ustekinumab

Of the 116 UC cases that initiated biologics between April 2019 and March 2022, 14 met the exclusion criteria. Thus, 102 cases were included in this study; 74 (72.5%) continued and 28 (27.5%) discontinued (Fig. 1). Table 1 shows the baseline characteristics of patients for each initiated biologic. There were significant differences in patient characteristics at the time of biologic initiation between the biologic agents. Compared with other agents, UST had a significantly higher proportion of patients with two or more prior biologic treatments (43.8% *vs*. 12.9%, *p* = 0.0016). Additionally, the proportion of patients with a history of anti-TNFα agents and VDZ was significantly higher in the UST group (anti-TNFα agents: 53.1% *vs*. 27.1%, *p* = 0.0144; VDZ: 37.5% *vs*. 7.1%, *p* = 0.0003). CRP levels were significantly higher in the IFX compared to other agents (*p* < 0.0001) and significantly lower in the VDZ (*p* = 0.0096). The partial Mayo score was significantly higher in the IFX and UST compared to other agents (IFX: *p* = 0.0064; UST: *p* = 0.0424) and significantly lower in the VDZ (*p* = 0.0141). The discontinuation rates for IFX, ADA, GLM, VDZ, and UST were 33.3%, 25.0%, 50.0%, 30.2%, and 15.6%, respectively (Fig. 2). Among the 28 patients who discontinued biologics, the most prevalent reason was lack of efficacy (85.7%), followed by adverse events (7.1%), pregnancy (3.6%), and death (3.6%). The reasons for discontinuation of each biologic are shown in Table 2.

**Table 1:** Baseline characteristics of patients for each initiated biologic

	IFX (*n* = 15)	ADA (*n* = 4)	GLM (*n* = 8)	VDZ (*n* = 43)	UST (*n* = 32)	*p*-value
Male	11 (73.3)	1 (25.0)	5 (62.5)	25 (58.1)	16 (50.0)	0.4288
Age^a^	41.0 (14–63)	33.5 (19–53)	41.0 (15–72)	34.0 (13–74)	36.5 (14–70)	0.9033
BMI^a^	18.3 (14.5–22.6)	22.0 (18.7–23.8)	20.9 (17.0–33.8)	20.0 (14.6–31.6)	20.9 (15.6–31.1)	0.1367
Disease duration (month)^a^	42.0 (3–377)	89.0 (23–313)	82.5 (7–259)	39.0 (2–360)	51.5 (8–404)	0.3383
Colonic area involved						0.4184
Pancolitis	15 (100)	4 (100)	7 (87.5)	35 (81.4)	28 (87.5)	
Left-sided colitis	0 (0.0)	0 (0.0)	1 (12.5)	8 (18.6)	4 (12.5)	
Concomitant medication						
Oral 5-ASA	9 (60.0)	4 (100)	6 (75.0)	28 (65.1)	22 (68.8)	0.7128
Topical 5-ASA	1 (6.7)	0 (0.0)	2 (25.0)	1 (2.3)	1 (3.1)	0.1229
Systemic corticosteroid	6 (40.0)	2 (50.0)	1 (12.5)	17 (39.5)	7 (21.9)	0.2695
Thiopurine	11 (73.3)	2 (50.0)	4 (50.0)	18 (41.9)	15 (46.9)	0.3345
Number of prior biologics						0.0297*
0	8 (53.3)	3 (75.0)	5 (62.5)	26 (60.5)	9 (28.1)	
1	6 (40.0)	1 (25.0)	1 (12.5)	11 (25.6)	9 (28.1)	
≥ 2	1 (6.7)	0 (0.0)	2 (25.0)	6 (14.0)	14 (43.8)	
Prior biologics therapies						
Anti-TNFα agents	1 (6.7)	1 (25.0)	3 (37.5)	14 (32.6)	17 (53.1)	0.0243*
VDZ	3 (20.0)	0 (0.0)	2 (25.0)	0 (0.0)	12 (37.5)	< 0.0001*
UST	0 (0.0)	0 (0.0)	0 (0.0)	0 (0.0)	1 (3.1)	0.5784
JAK inhibitors	4 (26.7)	0 (0.0)	0 (0.0)	3 (7.0)	6 (18.8)	0.1877
CRP^a^	3.00 (0.02–9.99)	0.15 (0.04–0.74)	0.34 (0.05–1.54)	0.25 (0.01–7.73)	0.70 (0.02–16.69)	0.0003*
pMayo score^a^	6.0 (0–8)	2.0 (0–7)	1.5 (0–7)	4.0 (0–8)	5.0 (0–9)	0.0021*

*BMI* Body mass index, *5-ASA* 5-aminosalicylates, *TNFα* Tumor necrosis factor α, *VDZ* Vedolizumab, *UST* Ustekinumab, *JAK inhibitors* Janus kinase inhibitors, *IFX* Infliximab, *ADA* Adalimumab, *GLM* Golimumab, *CRP* C-reactive protein, *pMayo score* Partial Mayo score

^a^median (range), all other values are in *N* (%)

^*^p < 0.05

**Table 2:** Reasons for discontinuation of biologics

	Infliximab	Adalimumab	Golimumab	Vedolizumab	Ustekinumab
Lack of efficacy	5	1	3	12	3
Adverse events	—	—	1	1	—
Pregnancy	—	—	—	—	1
Death	—	—	—	—	1
Total	5	1	4	13	5

### Factors associated with treatment discontinuation

Table 3 shows the demographic and clinical characteristics in the discontinuation and continuation groups. The discontinuation group had a significantly lower rate of concomitant thiopurine (28.6% *vs* 56.8%, *p* = 0.0132), and there was a trend toward fewer initiations with UST compared to the continuation group (17.9% *vs* 36.5%, *p* = 0.0769). No significant difference was observed in other baseline characteristics between the two groups. Based on these results, both concomitant thiopurine and the type of initiated biologic were incorporated as factors in the multivariate analysis. In the multivariable analysis, concomitant thiopurine was independently associated with continuation of biological therapy (*p* = 0.0075).

**Table 3:** Factors associated with biologics discontinuation

	Total (*n* = 102)	Continuation (*n* = 74)	Discontinuation (*n* = 28)	Univariate analysis			Multivariate analysis		
				OR	95% CI	*p*-value	AOR	95% CI	*p*-value
Male	58 (56.9)	44 (59.5)	14 (50.0)	0.68	(0.28–1.63)	0.3905			
Age^a^	37.5 (13–74)	39.0 (14–74)	35.0 (13–73)	1.00	(0.97–1.02)	0.9029			
BMI^a^	20.3 (14.5–33.8)	20.5 (14.6–33.8)	20.2 (14.5–29.5)	0.99	(0.88–1.11)	0.8283			
Disease duration (month)^a^	45.0 (2–404)	47.0 (2–404)	38.5 (2–224)	1.00	(0.99–1.00)	0.2771			
Colonic area involved						0.3075			
Pancolitis	89 (87.3)	63 (85.1)	26 (92.9)	2.27	(0.47–10.96)				
Left-sided colitis	13 (12.7)	11 (14.9)	2 (7.1)	1 (Ref)	—				
Concomitant medication									
Oral 5-ASA	69 (67.6)	50 (67.6)	19 (67.9)	1.01	(0.40–2.57)	0.9777			
Topical 5-ASA	5 (4.9)	3 (4.1)	2 (7.1)	1.82	(0.29–11.52)	0.5244			
Systemic corticosteroid	33 (32.4)	22 (29.7)	11 (39.3)	1.53	(0.62–3.79)	0.3588			
Thiopurine	50 (49.0)	42 (56.8)	8 (28.6)	0.30	(0.12–0.78)	0.0132*	0.25	(0.09–0.69)	0.0075*
Number of prior biologics						0.9030			
0	51 (50.0)	36 (48.6)	15 (53.6)	1 (Ref)	—				
1	28 (27.5)	21 (28.4)	7 (25.0)	0.80	(0.28–2.28)	0.6759			
≥ 2	23 (22.5)	17 (23.0)	6 (21.4)	0.85	(0.28–2.57)	0.7692			
Prior biologics therapies									
Anti-TNFα agents	36 (35.3)	25 (33.8)	11 (39.3)	1.27	(0.52–3.11)	0.6042			
VDZ	17 (16.7)	12 (16.2)	5 (17.9)	1.12	(0.36–3.54)	0.8428			
UST	1 (1.0)	1 (1.4)	0 (0.0)	—	—	0.9913			
JAK inhibitors	13 (12.7)	12 (16.2)	1 (3.6)	0.19	(0.02–1.55)	0.1209			
Initiated biologics						0.3509			0.2269
IFX	15 (14.7)	10 (13.5)	5 (17.9)			0.5816^+^	1 (Ref)	—	
ADA	4 (3.9)	3 (4.1)	1 (3.6)			0.9108^+^	0.45	(0.03–6.25)	0.5486
GLM	8 (7.8)	4 (5.4)	4 (14.3)			0.1511^+^	1.51	(0.23–9.75)	0.6628
VDZ	43 (42.2)	30 (40.5)	13 (46.4)			0.5913^+^	0.53	(0.13–2.10)	0.3662
UST	32 (31.4)	27 (36.5)	5 (17.9)			0.0769^+^	0.23	(0.05–1.08)	0.0628
CRP^a^	0.52 (0.01–16.69)	0.48 (0.02–16.69)	0.54 (0.01–4.34)	0.81	(0.60–1.09)	0.1681			
pMayo score^a^	5.0 (0–9)	5.0 (0–8)	5.0 (0–9)	1.03	(0.87–1.22)	0.7410			

There were no missing data; 102 cases were available for this analysis

*BMI* Body mass index, *5-ASA* 5-aminosalicylates, *TNFα* Tumor necrosis factor α, *VDZ* Vedolizumab, *UST* Ustekinumab, *JAK inhibitors* Janus kinase inhibitors, *IFX* Infliximab, *ADA* Adalimumab, *GLM* Golimumab, *CRP* C-reactive protein, *pMayo score* Partial Mayo score, *OR* Odds ratio, *CI* Confidence interval, *AOR* Adjusted odds ratio, *Ref* Reference

^a^median (range), all other values are in *N* (%)

^+^The statistical significance was examined for IFX, ADA, GLM, VDZ, and UST, each compared to others

^*^ *p* < 0.05

Supplementary tables 1–5 present the comparison of clinical characteristics between the discontinuation and continuation groups for each drug. In the case of IFX and UST, the discontinuation group had a significantly lower rate of concomitant thiopurine compared to the continuation group (IFX: 20.0% *vs*. 100%, *p* = 0.0037; UST: 0.0% *vs*. 55.6%, *p* = 0.0456).

### Concomitant use of thiopurines

**Fig. 3:**
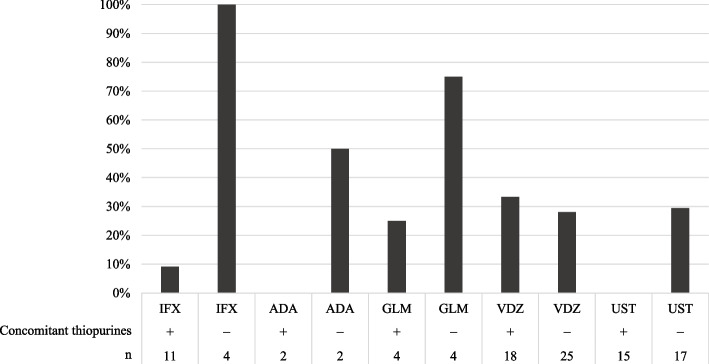
Discontinuation rate of each biologic with and without concomitant thiopurine. *IFX*, infliximab; *ADA*, adalimumab; *GLM*, golimumab; *VDZ*, vedolizumab; *UST*, ustekinumab

Discontinuation rates of each biologic were compared in relation to the concomitant use of thiopurine (Fig. 3). For IFX and UST, patients receiving concomitant thiopurine had a lower discontinuation rate than those not receiving concomitant thiopurine (IFX: 9.1% *vs*. 100%, *p* = 0.0037; UST: 0.0% *vs*. 29.4%, *p* = 0.0456).

## Discussion

This study sought to identify factors influencing therapeutic discontinuation by comparing the clinical characteristics of UC patients initiating biological therapy, grouped into continuation and discontinuation. Concomitant thiopurine use at the initiation of biologic therapy was significantly associated with a lower likelihood of treatment discontinuation, and UST initiation showed a tendency to reduce the risk of discontinuation. Multivariable analyses revealed that concomitant thiopurine was independently associated with the continuation of biological therapy.

Prior reports have shown that UC patients benefit more from a combination therapy of IFX and azathioprine than IFX monotherapy in achieving steroid-free remission induction and mucosal healing [15]. The combination of anti-TNF biologics and thiopurines is anticipated to reduce anti-drug antibody formation and prevent biologic response loss [16, 17]. This study found that the proportion of concomitant thiopurine use was significantly lower in the discontinuation group compared to the continuation group of biologics. It has been reported that thiopurine-induced adverse effects such as leukopenia and severe alopecia were associated with NUDT gene polymorphism [18, 19]. Of the 20 cases in discontinuation group who did not receive concomitant thiopurine therapy, 13 had NUDT15 Arg/Arg, 4 had Arg/Cys, and 3 had no data. Therefore, it is considered that the reason for the lower number of patients with concomitant thiopurine in the discontinuation group is not due to genetic polymorphisms. A comparison of discontinuation rates for each biological agent, with or without concomitant thiopurine, revealed lower rates in cases receiving concomitant thiopurine for IFX and UST. Hu et al. reported in a meta-analysis that combining VDZ or UST with thiopurine therapy did not increase rates of clinical remission, response, endoscopic remission, or one-year therapy persistence in patients with IBD [20]. However, this report included only four UC patients treated with UST, highlighting the need for further studies to evaluate the efficacy of UST and thiopurine combination therapy. This study found lower discontinuation rates for cases receiving UST with concomitant thiopurine, suggesting that concomitant thiopurine may aid in the continuation of UST therapy. However, as only five patients receiving UST were in the discontinuation group, further studies with larger cohorts are needed to enhance result reliability. Thus, except for IFX, there are no clear guidelines on the efficacy of combining biological agents with thiopurines, necessitating further research.

Moreover, cases initiating UST were less frequent in the discontinuation group. UST, the most recently approved among the five biologic agents studied, was initiated by a higher proportion of patients with prior biologic treatments compared to other agents (UST: 23 of 32; 71.9%, non-UST: 28 of 70; 40.0%, *p* = 0.0051). Generally, patients with prior biologic treatment have higher discontinuation rates [10, 21, 22]. However, this study found lower discontinuation rates among patients initiating UST, despite previous treatments with other biologics. UST, an anti-IL-12/23p40 antibody, may be effective in patients resistant to prior anti-TNFα antibody and anti-α_4_β_7_ integrin antagonist therapies. IL-23 upregulation in the mucosa of IBD patients resistant to anti-TNFα antibody and VDZ treatments [23, 24] may account for the low discontinuation rate despite high rates of prior biologic failure in patients initiating UST. Reports suggest that UST is more effective than VDZ for treatment retention and steroid-free remission rates in patients who previously failed anti-TNFα antibody therapies, although this was reported in Crohn’s disease [25, 26]. This study supports the reports. Although the present results showed only UST initiation was associated with a tendency to reduce treatment discontinuation, as shown in Table 1, the backgrounds of patients who initiated biologic differed, and to compare the discontinuation rate of each biologic, it is necessary to adjust for the patient backgrounds between the groups.

This study has several limitations, including its retrospective single-cohort design and small sample size, which limited the ability to perform subgroup analysis for each biological agent. In this study, patients who initiated biologic agents both as induction therapy and as maintenance therapy were included. To further investigate the factors associated with treatment discontinuation in each of these therapies, the accumulation of additional cases is required. Nevertheless, this study evaluated factors influencing therapeutic discontinuation across five biological agents, providing valuable insights for the continuation of biological therapy.

The concomitant use of thiopurines may positively impact the continuation of biologic therapy. Therefore, it is essential for physicians and pharmacists to support patients in maintaining thiopurine therapy safely and effectively. First, the NUDT15 genetic test should be performed to assess the suitability of thiopurine use. For patients prescribed thiopurines based on genetic test results, it is important to explain that it may take several weeks to months for the full therapeutic effect to become apparent, and to emphasize the importance of continuing treatment even if short-term benefits are not immediately observed. Therapy should be initiated at a low dose, with frequent blood tests to monitor leukocyte counts and liver function. Once the dose has stabilized at the maintenance level, regular testing every 3 to 6 months is necessary to detect potential side effects early. Additionally, a communication system should be established, enabling patients to consult with healthcare professionals promptly if they experience side effects or worsening symptoms. By providing these supports, physicians and pharmacists can help patients continue thiopurine therapy, thereby promoting the long-term continuation of biologic therapy.

In conclusion, our study suggests that concomitant thiopurine is a critical factor influencing the discontinuation of biological therapy in UC patients. However, further research is required to evaluate the efficacy of combination therapy with biologics and thiopurine.

## Supplementary Information

Additional file 1: Table S1. Baseline characteristics of patients treated with IFX. Table S2. Baseline characteristics of patients treated with ADA. Table S3. Baseline characteristics of patients treated with GLM. Table S4. Baseline characteristics of patients treated with VDZ. Table S5. Baseline characteristics of patients treated with UST.

## Data Availability

All data generated or analyzed during this study are included in this published article and its supplementary information files.

## Abbreviations

UC: Ulcerative colitis
IBD: Inflammatory bowel disease
5-ASA: 5-Aminosalicylic acid
IFX: Infliximab
ADA: Adalimumab
GLM: Golimumab
VDZ: Vedolizumab
UST: Ustekinumab
IBD: Inflammatory bowel disease
JAK: Janus kinase
CRP: C-reactive protein
