# Supplementary material for: Factors influencing the discontinuation of biologic therapies in patients with ulcerative colitis
Source: J Pharm Health Care Sci. 2024 Oct 18;10:65. doi: 10.1186/s40780-024-00386-2 (PMC11490000; doi:10.1186/s40780-024-00386-2)
Supplement: Supplementary file 1 — Additional file 1: Table S1. Baseline characteristics of patients treated with IFX. Table S2. Baseline characteristics of patients treated with ADA. Table S3. Baseline characteristics of patients treated with GLM. Table S4. Baseline characteristics of patients treated with VDZ. Table S5. Baseline characteristics of patients treated with UST. [file 40780_2024_386_MOESM1_ESM.docx]

Supplemental Table 1. Baseline characteristics of patients treated with infliximab (IFX)


|  | Total  (n = 15) | Continuation  (n = 10) | Discontinuation  (n = 5) | *p*-value |
| --- | --- | --- | --- | --- |
| Male | 11 (73.3) | 7 (70.0) | 4 (20.0) | 1.0000 |
| Age† | 41.0 (14–63) | 42.5 (19–63) | 25.0 (14–56) | 0.4620 |
| BMI† | 18.3 (14.5–22.6) | 19.5 (16.3–22.6) | 17.8 (14.5–20.0) | 0.0864 |
| Disease duration (month)† | 42.0 (3–377) | 42.5 (3–377) | 42.0 (3–224) | 0.8062 |
| Colonic area involved |  |  |  | 1.0000 |
| Pancolitis | 15 (100) | 10 (100) | 5 (100) |  |
| Left-sided colitis | 0 (0.0) | 0 (0.0) | 0 (0.0) |  |
| Concomitant medication |  |  |  |  |
| Oral 5-ASA | 9 (60.0) | 7 (70.0) | 2 (40.0) | 0.3287 |
| Topical 5-ASA | 1 (6.7) | 1 (10.0) | 0 (0.0) | 1.0000 |
| Systemic corticosteroid | 6 (40.0) | 4 (40.0) | 2 (40.0) | 1.0000 |
| Thiopurine | 11 (73.3) | 10 (100) | 1 (20.0) | 0.0037* |
| Number of prior biologics |  |  |  | 0.5338 |
| 0 | 8 (53.3) | 6 (60.0) | 2 (40.0) |  |
| 1 | 6 (40.0) | 4 (40.0) | 2 (40.0) |  |
| ≥ 2 | 1 (6.7) | 0 (0.0) | 1 (20.0) |  |
| Prior biologics therapies |  |  |  |  |
| Anti-TNFα agents | 1 (6.7) | 0 (0.0) | 1 (20.0) | 0.3333 |
| VDZ | 3 (20.0) | 0 (0.0) | 3 (60.0) | 0.0220* |
| UST | 0 (0.0) | 0 (0.0) | 0 (0.0) | 1.0000 |
| JAK inhibitors | 4 (26.7) | 3 (30.0) | 1 (20.0) | 1.0000 |
| CRP† | 3.00 (0.02–9.99) | 3.56 (0.02–9.99) | 1.50 (1.19–4.34) | 0.1113 |
| pMayo score† | 6.0 (0–8) | 6.0 (0–8) | 7.0 (5–8) | 0.3473 |

†: median (range), all other values are in N (%).

* *p* < 0.05

*BMI*, body mass index; *IFX*, infliximab; *5-ASA*, 5-aminosalicylates; *TNFα*, tumor necrosis factor α; *VDZ*, vedolizumab; *UST*, ustekinumab; *JAK inhibitors*, Janus kinase inhibitors; *ADA*, adalimumab; *GLM*, golimumab; *CRP*, C-reactive protein; *pMayo score*, partial Mayo score

Supplemental Table 2. Baseline characteristics of patients treated with adalimumab (ADA)


|  | Total  (n = 4) | Continuation  (n = 3) | Discontinuation  (n = 1) | *p*-value |
| --- | --- | --- | --- | --- |
| Male | 1 (25.0) | 1 (33.3) | 0 (0.0) | 1.0000 |
| Age† | 33.5 (19–53) | 43.0 (24–53) | 19.0 (19–19) | 0.1797 |
| BMI† | 22.0 (18.7–23.8) | 22.5 (18.7–23.8) | 21.5 (21.5–21.5) | 0.6547 |
| Disease duration (month)† | 89.0 (23–313) | 104.0 (74–313) | 23.0 (23–23) | 0.1797 |
| Colonic area involved |  |  |  | 1.0000 |
| Pancolitis | 4 (100) | 3 (100) | 1 (100) |  |
| Left-sided colitis | 0 (0.0) | 0 (0.0) | 0 (0.0) |  |
| Concomitant medication |  |  |  |  |
| Oral 5-ASA | 4 (100) | 3 (100) | 1 (100) | 1.0000 |
| Topical 5-ASA | 0 (0.0) | 0 (0.0) | 0 (0.0) | 1.0000 |
| Systemic corticosteroid | 2 (50.0) | 2 (66.7) | 0 (0.0) | 1.0000 |
| Thiopurine | 2 (50.0) | 2 (66.7) | 0 (0.0) | 1.0000 |
| Number of prior biologics |  |  |  | 1.0000 |
| 0 | 3 (75.0) | 2 (66.7) | 1 (100) |  |
| 1 | 1 (25.0) | 1 (33.3) | 0 (0.0) |  |
| ≥ 2 | 0 (0.0) | 0 (0.0) | 0 (0.0) |  |
| Prior biologics therapies |  |  |  |  |
| Anti-TNFα agents | 1 (25.0) | 1 (33.3) | 0 (0.0) | 1.0000 |
| VDZ | 0 (0.0) | 0 (0.0) | 0 (0.0) | 1.0000 |
| UST | 0 (0.0) | 0 (0.0) | 0 (0.0) | 1.0000 |
| JAK inhibitors | 0 (0.0) | 0 (0.0) | 0 (0.0) | 1.0000 |
| CRP† | 0.15 (0.04–0.74) | 0.10 (0.04–0.20) | 0.74 (0.74–0.74) | 0.1797 |
| pMayo score† | 2.0 (0–7) | 1.0 (0–3) | 7.0 (7–7) | 0.1797 |

†: median (range); all other values are in N (%).

*BMI*, body mass index; *ADA*, adalimumab; *5-ASA*, 5-aminosalicylates; *TNFα*, tumor necrosis factor α; *VDZ*, vedolizumab; *UST*, ustekinumab; *JAK inhibitors*, Janus kinase inhibitors; *IFX*, infliximab; *GLM*, golimumab; *CRP*, C-reactive protein; *pMayo score*, partial Mayo score

Supplemental Table 3. Baseline characteristics of patients treated with golimumab (GLM)


|  | Total  (n = 8) | Continuation  (n = 4) | Discontinuation  (n = 4) | *p*-value |
| --- | --- | --- | --- | --- |
| Male | 5 (62.5) | 4 (100) | 1 (25.0) | 0.1429 |
| Age† | 41.0 (15–72) | 41.0 (36–45) | 53.0 (15–72) | 0.7728 |
| BMI† | 20.9 (17.0–33.8) | 20.9 (18.0–33.8) | 21.1 (17.0–28.4) | 0.7728 |
| Disease duration (month)† | 82.5 (7–259) | 82.5 (7–259) | 95.5 (17–192) | 1.0000 |
| Colonic area involved |  |  |  | 1.0000 |
| Pancolitis | 7 (87.5) | 4 (100) | 3 (75.0) |  |
| Left-sided colitis | 1 (12.5) | 0 (0.0) | 1 (25.0) |  |
| Concomitant medication |  |  |  |  |
| Oral 5-ASA | 6 (75.0) | 3 (75.0) | 3 (75.0) | 1.0000 |
| Topical 5-ASA | 2 (25.0) | 0 (0.0) | 2 (50.0) | 0.4286 |
| Systemic corticosteroid | 1 (12.5) | 1 (25.0) | 0 (0.0) | 1.0000 |
| Thiopurine | 4 (50.0) | 3 (75.0) | 1 (25.0) | 0.4857 |
| Number of prior biologics |  |  |  | 0.4286 |
| 0 | 5 (62.5) | 2 (50.0) | 3 (75.0) |  |
| 1 | 1 (12.5) | 0 (0.0) | 1 (25.0) |  |
| ≥ 2 | 2 (25.0) | 2 (50.0) | 0 (0.0) |  |
| Prior biologics therapies |  |  |  |  |
| Anti-TNFα agents | 3 (37.5) | 2 (50.0) | 1 (25.0) | 1.0000 |
| VDZ | 2 (25.0) | 2 (50.0) | 0 (0.0) | 0.4286 |
| UST | 0 (0.0) | 0 (0.0) | 0 (0.0) | 1.0000 |
| JAK inhibitors | 0 (0.0) | 0 (0.0) | 0 (0.0) | 1.0000 |
| CRP† | 0.34 (0.05–1.54) | 0.32 (0.05–0.56) | 0.34 (0.27–1.54) | 0.5637 |
| pMayo score† | 1.5 (0–7) | 2.5 (0–5) | 1.5 (0–7) | 1.0000 |

†: median (range), all other values are in N (%).

*BMI*, body mass index; *GLM*, golimumab; *5-ASA*, 5-aminosalicylates; *TNFα*, Tumor necrosis factor α; *VDZ*, vedolizumab; *UST*, ustekinumab; *JAK inhibitors*, Janus kinase inhibitor; *IFX*, infliximab; *ADA*, adalimumab; *CRP*, C-reactive protein; *pMayo score*, partial Mayo score

Supplemental Table 4. Baseline characteristics of patients treated with vedolizumab (VDZ)


|  | Total  (n = 43) | Continuation  (n = 30) | Discontinuation  (n = 13) | *p-*value |
| --- | --- | --- | --- | --- |
| Male | 25 (58.1) | 17 (56.7) | 8 (61.5) | 1.0000 |
| Age† | 34.0 (13–74) | 37.0 (16–74) | 33.0 (13–73) | 0.3972 |
| BMI† | 20.0 (14.6–31.6) | 19.4 (14.6–31.6) | 20.8 (15.5–29.5) | 0.3411 |
| Disease duration (month)† | 39.0 (2–360) | 40.0 (2–360) | 35.0 (2–103) | 0.6818 |
| Colonic area involved |  |  |  | 0.3995 |
| Pancolitis | 35 (81.4) | 23 (76.7) | 12 (92.3) |  |
| Left-sided colitis | 8 (18.6) | 7 (23.3) | 1 (7.7) |  |
| Concomitant medication |  |  |  |  |
| Oral 5-ASA | 28 (65.1) | 18 (60.0) | 10 (76.9) | 0.4871 |
| Topical 5-ASA | 1 (2.3) | 1 (3.3) | 0 (0.0) | 1.0000 |
| Systemic corticosteroid | 17 (39.5) | 10 (33.3) | 7 (53.8) | 0.3098 |
| Thiopurine | 18 (41.9) | 12 (40.0) | 6 (46.2) | 0.7466 |
| Number of prior biologics |  |  |  | 0.3179 |
| 0 | 26 (60.5) | 20 (66.7) | 6 (46.2) |  |
| 1 | 11 (25.6) | 7 (23.3) | 4 (30.8) |  |
| ≥ 2 | 6 (14.0) | 3 (10.0) | 3 (23.1) |  |
| Prior biologics therapies |  |  |  |  |
| Anti-TNFα agents | 14 (32.6) | 7 (23.3) | 7 (53.8) | 0.0774 |
| VDZ | 0 (0.0) | 0 (0.0) | 0 (0.0) | 1.0000 |
| UST | 0 (0.0) | 0 (0.0) | 0 (0.0) | 1.0000 |
| JAK inhibitors | 3 (7.0) | 3 (10.0) | 0 (0.0) | 0.5418 |
| CRP† | 0.25 (0.01–7.73) | 0.25 (0.02–7.73) | 0.25 (0.01–2.28) | 0.6915 |
| pMayo score† | 4.0 (0–8) | 4.0 (0–8) | 3.0 (0–7) | 0.5199 |

†: median (range), all other values are in N (%).

*BMI*, body mass index; *VDZ*, vedolizumab; *5-ASA*, 5-aminosalicylates; *TNFα*, tumor necrosis factor α; *UST*, ustekinumab; *JAK inhibitors*, Janus kinase inhibitor; *IFX*, infliximab; *ADA*, adalimumab; *GLM*, golimumab; *CRP*, C-reactive protein; *pMayo score*, partial Mayo score

Supplemental Table 5. Baseline characteristics of patients treated with ustekinumab (UST)


|  | Total  (n = 32) | Continuation  (n = 27) | Discontinuation  (n = 5) | *p-*value |
| --- | --- | --- | --- | --- |
| Male | 16 (50.0) | 15 (55.6) | 1 (20.0) | 0.3326 |
| Age† | 36.5 (14–70) | 36.0 (14–68) | 43.0 (34–70) | 0.1254 |
| BMI† | 20.9 (15.6–31.1) | 20.9 (15.6–31.1) | 22.9 (19.0–24.5) | 0.3637 |
| Disease duration (month)† | 51.5 (8–404) | 49.0 (8–404) | 96.0 (14–135) | 0.6971 |
| Colonic area involved |  |  |  | 1.0000 |
| Pancolitis | 28 (87.5) | 23 (85.2) | 5 (100) |  |
| Left-sided colitis | 4 (12.5) | 4 (14.8) | 0 (0.0) |  |
| Concomitant medication |  |  |  |  |
| Oral 5-ASA | 22 (68.8) | 19 (70.4) | 3 (60.0) | 0.6367 |
| Topical 5-ASA | 1 (3.1) | 1 (3.7) | 0 (0.0) | 1.0000 |
| Systemic corticosteroid | 7 (21.9) | 5 (18.5) | 2 (40.0) | 0.2964 |
| Thiopurine | 15 (46.9) | 15 (55.6) | 0 (0.0) | 0.0456* |
| Number of prior biologics |  |  |  | 0.1459 |
| 0 | 9 (28.1) | 6 (22.2) | 3 (60.0) |  |
| 1 | 9 (28.1) | 9 (33.3) | 0 (0.0) |  |
| ≥ 2 | 14 (43.8) | 12 (44.4) | 2 (40.0) |  |
| Prior biologics therapies |  |  |  |  |
| Anti-TNFα agents | 17 (53.1) | 15 (55.6) | 2 (40.0) | 0.6454 |
| VDZ | 12 (37.5) | 10 (37.0) | 2 (40.0) | 1.0000 |
| UST | 1 (3.1) | 1 (3.7) | 0 (0.0) | 1.0000 |
| JAK inhibitors | 6 (18.8) | 6 (22.2) | 0 (0.0) | 0.5546 |
| CRP† | 0.70 (0.02–16.69) | 0.75 (0.03–16.69) | 0.52 (0.02–3.08) | 0.7753 |
| pMayo score† | 5.0 (0–7) | 5.0 (0–9) | 6.0 (5–9) | 0.0721 |

†: median (range), all other values are in N (%).

* *p* < 0.05

*BMI*, body mass index; *UST*, ustekinumab; *5-ASA*, 5-aminosalicylates; *TNFα*, tumor necrosis factor α; *VDZ*, vedolizumab; *JAK inhibitors*, Janus kinase inhibitor; *IFX*, infliximab; *ADA*, adalimumab; *GLM*, golimumab; *CRP*, C-reactive protein; *pMayo score*, partial Mayo score
